# Effect of Stabilization Exercises Added to Routine Physiotherapy on Trunk Muscle Dimensions, Pain, Disability, and Lumbar Range of Motion in Patients With Unilateral Lumbar Disc Protrusion: A Randomized Controlled Trial

**DOI:** 10.1002/hsr2.72864

**Published:** 2026-07-25

**Authors:** Morteza Taghipour, Nahid Rahmani, Mohammad Ali Mohseni Bandpei, Fatemeh Rajabzadeh, Iraj Abdollahi, Azadehsadat Mirtaleb

**Affiliations:** ^1^ Neuromusculoskeletal Rehabilitation Research Center University of Social Welfare and Rehabilitation Sciences Tehran Iran; ^2^ University Institute of Physical Therapy, Faculty of Allied Health Sciences, University of Lahore Lahore Pakistan; ^3^ School of Health, Psychological, and Medical Sciences University of Southern Queensland Toowoomba Australia

**Keywords:** functional disability, lumbar disc protrusion, pain intensity, stabilization exercises, trunk muscle dimensions, ultrasonography

## Abstract

**Background and Aims:**

To investigate the effects of stabilization exercises added to routine physiotherapy on trunk muscle dimensions, pain intensity, functional disability, and lumbar spine range of motion (ROM) in patients with unilateral lumbar disc protrusion.

**Methods:**

This randomized assessor‐blinded controlled clinical trial was conducted in an outpatient physiotherapy clinic. Sixty patients diagnosed with unilateral lumbar disc protrusion were randomly allocated to an experimental or control group. The control group received routine physiotherapy, while the experimental group received routine physiotherapy combined with supervised stabilization exercises. Trunk muscle dimensions, including thickness of the deep abdominal muscles (transversus abdominis, internal oblique, and external oblique), psoas major, and lumbar multifidus, were assessed using ultrasonography at rest and during contraction. Pain intensity, functional disability, and lumbar spine ROM were also evaluated before and after the intervention. Changes in muscle thickness during contraction were evaluated as indicators of muscle performance.

**Results:**

Both groups showed significant improvements in pain intensity, functional disability, and lumbar spine ROM (*p* < 0.05). However, the experimental group demonstrated significantly greater improvements in pain intensity and lumbar spine ROM. In addition, muscle thickness during contraction changed significantly in the experimental group (*p* < 0.05). These changes are interpreted as indirect indicators of potential functional or neuromuscular activation changes rather than definitive structural adaptations.

**Conclusion:**

Adding stabilization exercises to routine physiotherapy was associated with improvements in pain, lumbar mobility, and trunk muscle thickness in patients with unilateral lumbar disc protrusion, with potential clinical implications for optimizing conservative physiotherapy programs.

**Abbreviation**
ROM: range of motion

## 1 Introduction

Low back pain is one of the most common musculoskeletal disorders worldwide and represents a leading cause of disability across different age groups [1]. Lumbar disc protrusion is a frequent underlying cause of low back pain and may result in pain, functional limitations, and reduced quality of life [2, 3, 4, 5].

Previous studies have reported alterations in trunk muscle dimensions in individuals with lumbar disc protrusion, particularly involving the lumbar multifidus, deep abdominal muscles, and psoas major [6, 7, 8]. However, the extent and clinical relevance of these changes remain incompletely understood.

In recent years, stabilization exercises have been increasingly incorporated into conservative management approaches for patients with lumbar disc protrusion to target trunk muscle control and spinal stability [9]. Previous studies have suggested that stabilization exercise programs may lead to improvements in pain and functional outcomes in this population [10, 11, 12].

Despite the growing use of stabilization exercises in the conservative management of lumbar disc protrusion, limited evidence exists regarding their specific effects on trunk muscle thickness, particularly when assessed by ultrasonography. In addition, few studies have simultaneously examined dimension changes in key trunk muscles alongside clinical outcomes such as pain, functional disability, and lumbar spine range of motion (ROM).

Therefore, this study aimed to investigate the effects of stabilization exercises added to routine physiotherapy on trunk muscle dimensions, pain intensity, functional disability, and lumbar spine ROM in patients with unilateral lumbar disc protrusion, with potential clinical implications for optimizing conservative physiotherapy programs.

## 2 Methods

### 2.1 Study Design

This randomized, assessor‐blinded controlled clinical trial ensured blinding of the outcome assessors. Due to the nature of the intervention, blinding of the treating physiotherapist and participants was not feasible. The study protocol was approved by the Ethics Committee of the University of Social Welfare and Rehabilitation Sciences, and all participants provided written informed consent. The study was conducted in accordance with the Declaration of Helsinki.

### 2.2 Changes in Methods and Outcomes

No major changes were made during the study. Minor adjustments, such as reminder calls to improve home exercise compliance, were implemented without affecting study integrity.

### 2.3 Participants

Participants were recruited from patients referred to the outpatient physiotherapy clinic. Eligible participants were adults diagnosed with unilateral lumbar disc protrusion at the L4–L5 or L5–S1 level. Diagnosis was confirmed based on magnetic resonance imaging (MRI) findings in conjunction with clinical evaluation by a spinal surgeon.

Only patients with ongoing symptoms lasting longer than 3 months were included to ensure that participants were not in the acute phase and did not present with recurrent episodic symptoms. Patients with bilateral disc involvement, previous lumbar spine surgery, spinal fractures, inflammatory spinal disorders, or other neurological conditions were excluded from the study.

Participants were randomly allocated to either the experimental or control group using a computer‐generated randomization sequence. Group allocation was concealed using sealed opaque envelopes. Outcome assessors were blinded to group allocation. However, due to the nature of the interventions, neither the treating physiotherapist nor the participants could be blinded. However, because the active nature of the physical exercises precluded true participant and therapist blinding, this limitation may have inherently introduced expectation bias among participants or performance bias among treating clinicians. To mitigate these risks, participants were kept strictly unaware of the specific study hypotheses, and highly standardized treatment protocols were enforced across both groups; nevertheless, the postintervention outcomes must be interpreted with appropriate caution regarding these potential biases.

### 2.4 Sample Size Determination

The sample size was calculated using GPower software (version 3.1) to ensure sufficient power for detecting clinically meaningful differences in the primary outcome (pain intensity, measured by the Visual Analog Scale) and secondary outcomes (muscle thickness, functional disability, and lumbar ROM). A priori power analysis was conducted with a significance level of 0.05 and a statistical power of 80% (1‐*β* = 0.80). The effect size (Cohen's *d*) was estimated based on previous studies evaluating the effectiveness of stabilization exercises in patients with lumbar disc protrusion and low back pain. The analysis determined that a minimum of 24 participants per group (total *N* = 48) would be required. To account for potential dropouts and maintain statistical power, the sample size was increased to 30 participants per group (total *N* = 60). This adjustment accounted for an anticipated dropout rate of approximately 20%. A statistician reviewed and approved the calculation process and assumptions before initiating the study.

### 2.5 Procedure

The ultrasound machine used in this study was the ES500 model from Ultrasonix, Canada. All ultrasound measurements were obtained using a B‐mode ultrasound system equipped with a 5 MHz convex probe [13]. It should be noted that the recording of images and measurement of muscle dimensions were conducted by the first and second therapists, respectively, with the second therapist unaware of whether the patients were in the experimental or control group.

#### 2.5.1 Abdominal Muscle Measurement

**Figure 1:**
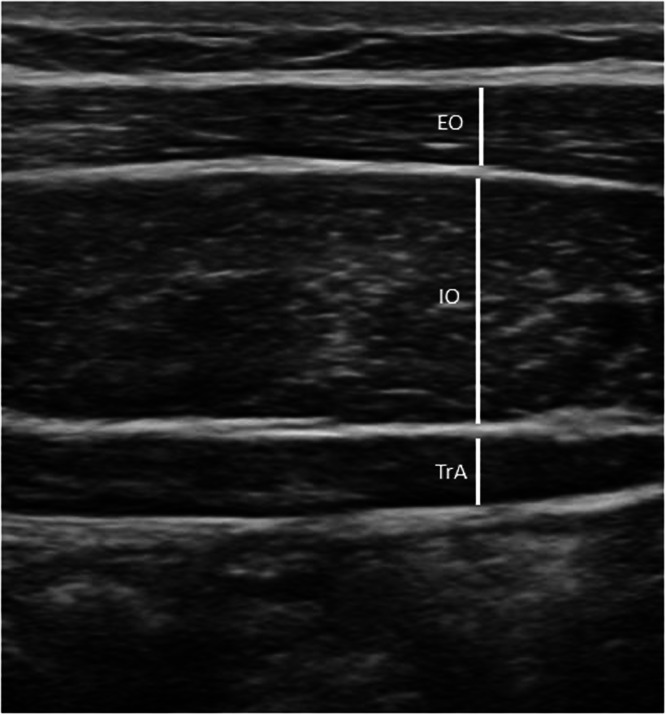
Ultrasound imaging of the deep abdominal muscles during assessment, demonstrating the transversus abdominis, internal oblique, and external oblique layers.

Abdominal muscle thickness was measured with participants in a supine, knees‐bent position. Images were captured bilaterally during rest and contraction, the latter using abdominal hollowing with pressure biofeedback (from 20 to 40 mmHg) [14, 15]. The probe was placed mid‐laterally along the mid‐axillary line, ~2.5 cm anterior to the midpoint between the iliac crest and 12th rib, with measurements 2 cm lateral to the TrA origin. Images were captured at end‐expiration, and participants could not view the screen [16, 17, 18] (Figure 1).

#### 2.5.2 Multifidus Muscle Measurement

**Figure 2:**
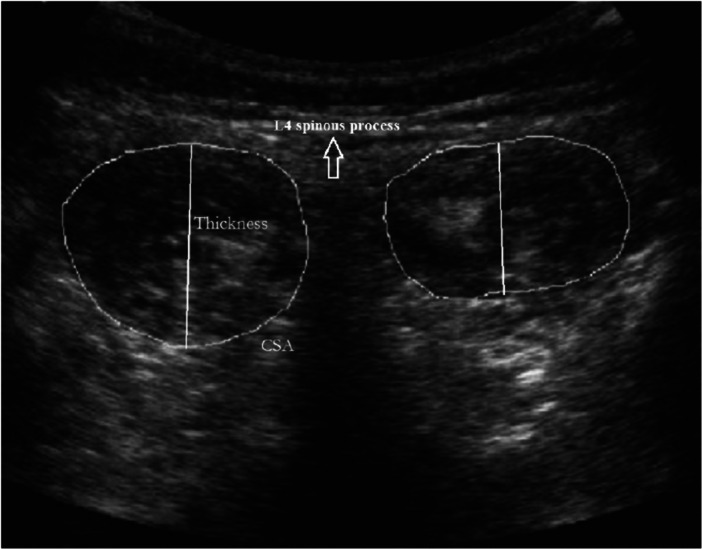
Bilateral ultrasound imaging of the lumbar multifidus muscle at the L4 vertebral level during measurement.

For multifidus measurement, participants lie prone with a pillow under the abdomen to reduce lumbar lordosis (< 10°, measured via inclinometer). The probe was positioned vertically at L4–L5 and L5–S1 to assess CSA and AP thickness, using anatomical landmarks: thoracolumbar fascia and lamina (superior/inferior boundaries), spinous process shadow (medial), and separating fascia (lateral) [19]. AP thickness was calculated as the distance between the superior and inferior boundaries [20, 21]. Changes in muscle thickness during contraction were evaluated using a prone double‐arm lift (~1 cm above the surface), with imaging at end‐expiration and screen visibility blocked (Figure 2).

#### 2.5.3 Psoas Major Muscle Measurement

**Figure 3:**
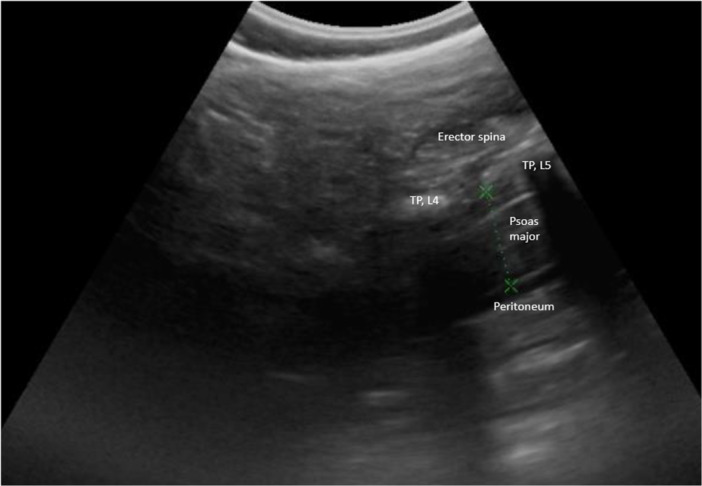
Longitudinal ultrasound imaging of the psoas major muscle at the L4–L5 level during thickness assessment.

Participants were positioned seated on a chair with their head, cervical spine, and trunk in a neutral position. Lumbar spinous processes were palpated manually and marked on the skin. The location of the spinous processes was confirmed using ultrasound imaging in the parasagittal plane, referencing the sacrum bone. The ultrasound probe was placed longitudinally 3–4 cm lateral to the spinous processes at the L4–L5 level to visualize the acoustic shadows of the transverse processes. The thickness of the psoas major muscle was measured by determining the distance between the deep fascia of the psoas major and the abdominal fascia [22] (Figure 3).

All ultrasound image acquisition and measurements were performed by an experienced rater with more than 10 years of experience in spine imaging analysis. The assessors received training from a senior musculoskeletal ultrasound radiologist prior to the start of the study. To minimize measurement error, the average of three images was used for analysis.

### 2.6 Intervention

Group allocation procedures are described in the Participants section. Allocation was concealed via sealed envelopes prepared by an independent staff member. Outcome assessors were blinded to group allocation to minimize bias.

The study was conducted at the University of Social Welfare and Rehabilitation Sciences, Tehran, Iran. Assessments and supervised sessions were held in the lab; home exercise adherence was monitored via phone calls.

The control group received routine physiotherapy, including Infra‐red (20 min), TENS (150 Hz, 80 µs pulse duration, 20 min) [23], and continuous ultrasound (5 cm^2^ applicator, 1 MHz, 1.5 W/cm^2^, 5–8 min) to the lumbar paraspinal muscles [24, 25] along with neuromobilization exercises. The experimental group received the same routine physiotherapy, in addition to a specific stabilization exercise program designed to target the deep trunk muscles, including the multifidus and transverse abdominis. This program, based on the Koumantakis protocol, aimed to improve trunk stability and pain‐related outcomes [26].

Both groups participated in therapy sessions three times per week for 8 weeks (24 sessions in total, with 1 day in between) under the supervision of a physiotherapist with over 5 years of experience. Patients were also instructed to perform the exercises on the other days of the week according to a notebook provided to them. To encourage adherence, phone calls were made to remind patients to complete their exercises at home. Progression through the exercise program was individualized based on each patient's response and tolerance.

At the end of the 8‐week intervention period, all participants underwent a reassessment of all baseline measures. Only patients who completed at least 75% of the treatment sessions and reached the final stage of the stabilization exercises were included in the final analysis.

### 2.7 Statistical Analysis

All collected data were analyzed using SPSS software (version 24.0; IBM Corp., Armonk, NY, USA). The Kolmogorov–Smirnov test was used to assess the normality of data distribution. An independent *t*‐test was applied to compare mean values between groups, while a paired *t*‐test was used to compare measurements before and after the intervention within each group. A significance level of *p* < 0.05 was considered statistically significant [27]. All statistical tests were two‐sided. All statistical analyses were prespecified in the study protocol, and no exploratory or subgroup analyses were conducted. No interim analyses or stopping guidelines were applied in this study. Although this approach was prespecified, the use of multiple *t*‐tests inherently increases the risk of type I error inflation and precludes formal group‐by‐time interaction testing. To mitigate this risk without altering the preregistered protocol, interpretations of the secondary ultrasonographic outcomes were conducted with strict caution, conceptually prioritizing highly significant changes (*p* ≤ 0.01) as robust findings, while treating borderline significant values (e.g., *p* = 0.03 or 0.04) with clinical reservation.

## 3 Results

**Figure 4:**
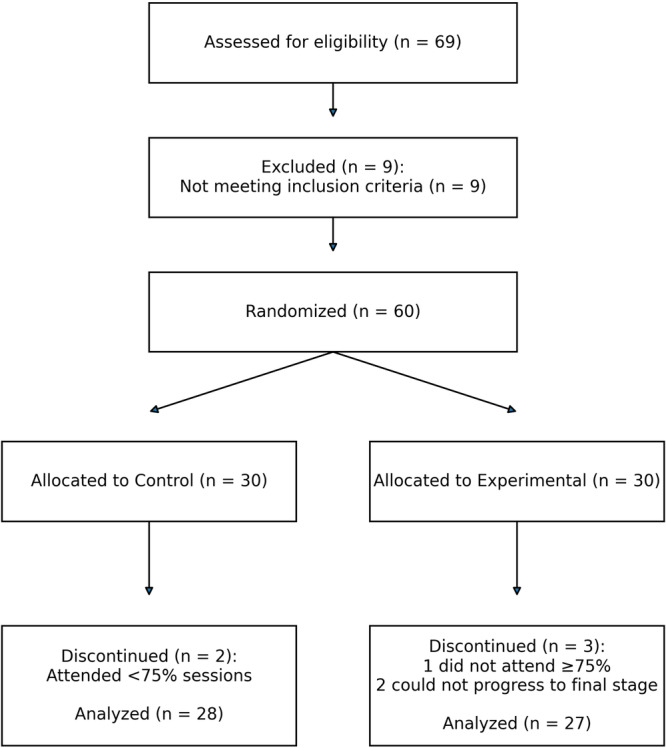
CONSORT flow diagram illustrating participant recruitment, eligibility assessment, randomization, allocation, follow‐up, and analysis.

Thirty participants were initially allocated to each group. In the control group, two participants were excluded due to attending fewer than 18 out of 24 treatment sessions. In the experimental group, one participant was excluded for insufficient attendance, and two participants were excluded due to inability to progress to the final stage of the stabilization exercise program. Ultimately, data from 28 participants in the control group and 27 participants in the experimental group were included in the final analysis (Figure 4).

No serious adverse effects were reported in either group during the intervention period. Mild muscle soreness was reported by a few participants in the experimental group during the initial weeks, which resolved spontaneously. No participant experienced worsening of symptoms or treatment‐related injuries.

The Kolmogorov–Smirnov test confirmed normal distribution of all variables, supporting the use of parametric tests.

Independent *t*‐tests showed no significant differences between the two groups at baseline in demographic characteristics, trunk muscle dimensions, or lumbar spine ROM (*p* > 0.05; Table 1). This finding indicates that the groups were well‐matched at baseline, minimizing the influence of confounding factors on the study outcomes.

**Table 1:** Comparison of demographic variables and muscle dimensions between the control and the experimental groups at baseline.

Variable	Group	Mean	SD	*t*‐statistic	DF	*p*‐value	95% CI (Lower–Upper)
Age (years)	Control	41.50	11.94	0.20	53	**0.84**	−5.35 to 6.55
	Experimental	40.90	11.09				
Height (cm)	Control	168.03	8.40	0.57	53	**0.57**	−3.25 to 5.85
	Experimental	166.73	8.17				
Weight (kg)	Control	69.66	10.91	0.99	53	**0.32**	−3.04 to 9.04
	Experimental	66.66	10.43				
BMI (kg/m^2^)	Control	24.66	3.69	0.64	53	**0.52**	−1.34 to 2.60
	Experimental	23.82	3.93				
Pain Level	Control	5.70	1.46	0.93	53	**0.44**	−0.21 to 1.28
	Experimental	5.16	1.41				
Functional Disability	Control	54.60	12.57	0.69	53	**0.49**	−4.04 to 8.31
	Experimental	52.46	11.30				
ROM: Flexion (°)	Control	35.41	10.52	0.72	53	**0.41**	−1.29 to 3.42
	Experimental	36.08	10.36				
ROM: Extension (°)	Control	14.45	4.11	0.66	53	**0.51**	−2.03 to 2.79
	Experimental	14.72	3.69				
TrA (mm, healthy side)	Control	3.32	0.75	0.52	53	**0.60**	−0.27 to 0.47
	Experimental	3.22	0.71				
TrA (mm, affected side)	Control	2.63	0.69	0.32	53	**0.74**	−0.29 to 0.39
	Experimental	2.57	0.63				
IO (mm, healthy side)	Control	5.25	1.28	1.35	53	**0.18**	−0.22 to 0.16
	Experimental	4.78	1.48				
IO (mm, affected side)	Control	4.71	1.10	0.21	53	**0.83**	−0.63 to 0.51
	Experimental	4.77	1.11				
EO (mm, healthy side)	Control	3.54	1.48	0.86	53	**0.39**	−0.43 to 1.07
	Experimental	2.85	1.43				
EO (mm, affected side)	Control	3.54	1.22	0.35	53	**0.72**	−0.80 to 0.56
	Experimental	3.66	1.40				
MF CSA (cm^2^, healthy side)	Control	4.18	0.95	0.16	53	**0.87**	−0.43 to 0.50
	Experimental	4.14	0.84				
MF CSA (cm^2^, affected side)	Control	3.87	1.01	0.27	53	**0.78**	−0.45 to 0.59
	Experimental	3.80	0.99				
Psoas (mm, healthy side)	Control	33.08	3.81	0.13	53	**0.89**	−1.59 to 1.81
	Experimental	32.97	2.67				
Psoas (mm, affected side)	Control	30.60	3.26	0.27	53	**0.79**	−1.28 to 1.68
	Experimental	30.40	2.41				

*Note:* Bold values indicate statical significant.

Abbreviations: 95% CI, 95% confidence interval; BMI, body mass index; DF, degrees of freedom; EO, external oblique; IO, internal oblique; MF CSA, multifidus cross sectional area; ROM, range of motion; SD, standard deviation; TrA, transversus abdominis.

For within‐group comparisons, mean differences were calculated as baseline minus postintervention values (pre–post). Therefore, negative values indicate an increase after treatment, whereas positive values indicate a decrease.

Paired *t*‐tests revealed significant improvements in pain intensity, functional disability, and lumbar ROM in both groups following 8 weeks of intervention (*p* < 0.05). The magnitude of improvement was greater in the experimental group for pain intensity (2.20 ± 1.58 vs. 1.37 ± 1.54), disability (18.23 ± 6.95 vs. 7.66 ± 11.82), lumbar flexion ROM (−11.83° ± 5.70 vs. −7.21° ± 5.74), and lumbar extension ROM (−8.36° ± 3.09 vs. −5.16° ± 2.66) (Table 2).

**Table 2:** Paired *t*‐test results for mean differences before and after treatment in the control and the experimental groups.

Variable	Group	Mean difference (pre–post)	SD	*t*‐statistic	*p*‐value	95% CI (lower–upper)
Pain intensity	Control	1.37	1.54	4.85	< 0.001	0.79–1.94
	Experimental	2.20	1.58	7.60	< 0.001	1.61–2.79
Functional disability	Control	7.66	11.82	3.37	0.02	2.85–11.68
	Experimental	18.23	6.95	14.37	< 0.001	15.64–20.82
Flexion ROM (°)	Control	−7.21	5.74	3.16	0.01	−11.89 to −2.53
	Experimental	−11.83	5.70	11.56	< 0.001	−13.93 to −9.73
Extension ROM (°)	Control	−5.16	2.66	3.23	0.01	−8.44 to −1.88
	Experimental	−8.36	3.09	5.76	< 0.001	−11.34 to −5.38

Abbreviations: 95% CI, 95% confidence interval; ROM, range of motion; SD, standard deviation.

In the control group, no significant changes were observed in muscle thickness at rest (*p* = 0.07–0.75). During contraction, significant changes were observed in the transversus abdominis (TrA), internal oblique (IO), and Multifidus (MF) cross‐sectional area on the affected side (Table 3). A borderline significant increase in MF thickness was also observed on the affected side after 8 weeks of routine physiotherapy (mean difference = −1.73 ± 4.28 mm, *p* = 0.03; Table 3).

**Table 3:** Paired *t*‐test results for the mean difference in muscle dimensions before and after treatment in the control group at rest and during contraction.

Variable	Side	Status	Mean difference (pre–post) (SD)	*t*‐statistic (*p*‐value)	95% CI (lower–upper)
TrA (mm)	Healthy	Rest	−0.12 (1.20)	−0.58 (0.57)	−0.57 to 0.32
		Contraction	0.12 (0.66)	1.00 (0.32)	−0.37 to 0.13
	Affected	Rest	−0.12 (0.67)	−1.00 (0.32)	−0.37 to 0.13
		Contraction	0.54 (1.27)	2.33 (0.02)*	0.07 to 1.02
IO (mm)	Healthy	Rest	−0.59 (2.16)	−1.49 (0.14)	−1.40 to 0.22
		Contraction	−0.49 (1.45)	−1.89 (0.07)	−1.04 to 0.04
	Affected	Rest	−0.50 (1.45)	−1.89 (0.07)	−1.04 to 0.04
		Contraction	0.56 (1.23)	2.50 (0.02)*	0.01 to 1.02
EO (mm)	Healthy	Rest	0.31 (1.82)	0.93 (0.36)	−0.99 to 0.37
		Contraction	0.21 (0.78)	1.51 (0.14)	−0.51 to 0.08
	Affected	Rest	0.21 (0.78)	1.50 (0.14)	−0.50 to 0.08
		Contraction	0.29 (1.01)	1.55 (0.13)	−0.66 to 0.09
MF‐CSA (cm^2^)	Healthy	Rest	−0.08 (1.36)	−0.32 (0.75)	−0.59 to 0.43
		Contraction	−0.27 (1.22)	−1.21 (0.23)	−0.73 to 0.18
	Affected	Rest	−0.27 (1.22)	−1.21 (0.23)	−0.73 to 0.18
		Contraction	0.61 (1.45)	2.29 (0.03)*	0.01 to 1.15
MF Thickness (mm)	Healthy	Rest	−0.45 (3.87)	−0.64 (0.53)	−1.90 to 0.99
		Contraction	−1.42 (3.84)	−2.03 (0.05)	−2.86 to 0.01
	Affected	Rest	−1.42 (3.84)	−2.03 (0.05)	−2.86 to 0.01
		Contraction	−1.73 (4.28)	−2.21 (0.03)*	−3.32 to −1.13
Psoas (mm)	Healthy	Rest	−0.83 (4.48)	−1.02 (0.32)	−2.50 to 0.84
	Affected	Rest	−1.04 (4.26)	−1.33 (0.19)	−2.63 to 0.55

Abbreviations: 95% CI, 95% confidence interval; EO, external oblique; IO, internal oblique; MF CSA, multifidus cross‐sectional area; SD, standard deviation; TrA, transversus abdominis.

* Significant at *p* < 0.05.

In the experimental group, statistically significant changes (*p* < 0.05) were observed in muscle thickness during contraction for most assessed muscles on both the affected and unaffected sides, except for the external oblique (*p* = 0.60–0.98) (Table 4).

**Table 4:** Paired *t*‐test results for the mean difference in muscle dimensions before and after treatment in the experimental group at rest and during contraction.

Variable	Side	Status	Mean difference (Pre−post) (SD)	*t‐s*tatistic (*p*‐value)	95% CI (lower–upper)
TrA (mm)	Healthy	Rest	−0.37 (0.92)	−2.23 (0.03)*	−0.72 to −0.03
		Contraction	−0.99 (1.39)	−3.23 (< 0.001)*	−1.53 to −0.47
	Affected	Rest	−0.61 (0.95)	−3.52 (< 0.001)*	−0.97 to −0.25
		Contraction	−1.25 (1.42)	−5.42 (< 0.001)*	−1.78 to −0.71
IO (mm)	Healthy	Rest	−0.89 (2.26)	−2.17 (0.04)*	−1.74 to −0.05
		Contraction	−1.21 (2.29)	−3.61 (< 0.001)*	−1.53 to −0.05
	Affected	Rest	−0.83 (1.35)	−3.38 (< 0.001)*	−1.34 to −0.33
		Contraction	−1.44 (1.96)	−4.54 (< 0.001)*	−2.34 to −0.71
EO (mm)	Healthy	Rest	−0.18 (1.88)	−0.53 (0.60)	−0.88 to 0.59
		Contraction	−0.17 (0.53)	−0.53 (0.53)	−0.88 to 0.39
	Affected	Rest	0.00 (1.78)	−0.02 (0.98)	−0.67 to 0.66
		Contraction	−0.07 (0.71)	−0.22 (0.82)	−0.71 to 0.57
MF‐CSA (cm^2^)	Healthy	Rest	−0.49 (1.21)	−2.23 (0.03)*	−0.94 to −0.04
		Contraction	−0.70 (1.21)	−2.86 (0.01)*	−1.94 to −0.04
	Affected	Rest	−1.08 (1.08)	−3.75 (< 0.001)*	−1.98 to −0.17
		Contraction	−1.08 (1.08)	−3.72 (< 0.001)*	−1.98 to −0.13
MF thickness (mm)	Healthy	Rest	−1.23 (3.62)	−2.86 (0.07)	−2.58 to 0.12
		Contraction	−2.86 (4.62)	−2.75 (0.03)*	−3.82 to −0.12
	Affected	Rest	−3.79 (3.59)	−2.75 (0.01)*	−4.14 to −0.46
		Contraction	−2.02 (5.92)	−3.75 (< 0.001)*	−4.55 to −0.80
Psoas (mm)	Healthy	Rest	−1.30 (4.08)	−1.75 (0.09)	−2.83 to 0.22
	Affected	Rest	−2.81 (4.74)	−2.13 (0.01)*	−3.62 to −0.08

Abbreviations: 95% CI, 95% confidence interval; EO, external oblique; IO, internal oblique; MF CSA, multifidus cross‐sectional area; SD, standard deviation; TrA, transversus abdominis.

* Significant at *p* < 0.05.

Between‐group comparisons using independent *t*‐tests demonstrated significant differences after the intervention in muscle dimensions on the affected side (*p* = 0.01–0.04, Tables 5 and 6), pain intensity (*p* < 0.001), lumbar ROM (*p* < 0.01), and functional disability (*p* < 0.001, Table 7). These preliminary between‐group comparisons should be interpreted with appropriate caution, descriptive of co‐existing clinical trends rather than definitive group‐by‐time interactions.

**Table 5:** Independent *t*‐test results for mean differences in muscle dimensions between the experimental and the control groups at rest on healthy and affected sides.

Muscle dimension	Side of body	Group	Mean (SD)	*t*‐statistic	*p*‐value	95% CI (lower–upper)
TrA (mm)	Healthy	Control	3.45 (0.78)	−0.70	0.49	−0.57 to 0.27
		Experimental	3.60 (0.88)			
	Affected	Control	2.75 (0.75)	−2.46	0.01*	−0.78 to −0.08
		Experimental	3.19 (0.61)			
IO (mm)	Healthy	Control	5.84 (1.72)	0.40	0.69	−0.99 to 0.66
		Experimental	5.67 (1.49)			
	Affected	Control	5.21 (1.88)	−2.05	0.04*	−1.18 to −0.38
		Experimental	5.60 (1.02)			
EO (mm)	Healthy	Control	3.23 (1.21)	−0.57	0.57	−0.42 to 0.76
		Experimental	3.03 (1.08)			
	Affected	Control	3.32 (1.47)	−1.07	0.28	−0.29 to 0.97
		Experimental	3.67 (0.91)			
MF‐CSA (cm^2^)	Healthy	Control	4.26 (0.85)	−1.64	0.10	−0.83 to 0.08
		Experimental	4.64 (0.91)			
	Affected	Control	4.14 (0.98)	−2.05	0.04*	−1.69 to −0.21
		Experimental	4.88 (0.78)			
MF Thickness (mm)	Healthy	Control	22.31 (2.66)	−0.04	0.98	−1.53 to 1.47
		Experimental	22.34 (3.14)			
	Affected	Control	21.14 (2.61)	−2.38	0.02*	−2.91 to −0.25
		Experimental	22.72 (2.80)			
Psoas (mm)	Healthy	Control	33.91 (3.00)	−0.45	0.65	−1.96 to 1.24
		Experimental	34.27 (3.20)			
	Affected	Control	31.63 (3.01)	−2.08	0.04*	−2.44 to −1.22
		Experimental	33.24 (4.00)			

Abbreviations: 95% CI, 95% confidence interval; EO, external oblique; IO, internal oblique; MF CSA, multifidus cross‐sectional area; SD, standard deviation; TrA, transversus abdominis.

* Significant at *p* < 0.05.

**Table 6:** Independent *t*‐test results for mean differences in muscle dimensions between the experimental and the control groups during contraction on healthy and affected sides.

Muscle dimension	Side of body	Group	Mean (SD)	*t*‐statistic	*p*‐value	95% CI (lower–upper)
TrA (mm)	Healthy	Control	5.28 (1.31)	0.49	0.62	−0.83 to 0.50
		Experimental	5.12 (1.27)			
	Affected	Control	3.19 (0.72)	−2.25	0.01*	−0.63 to −0.32
		Experimental	4.24 (0.74)			
IO (mm)	Healthy	Control	7.47 (2.05)	0.36	0.72	−0.77 to 1.11
		Experimental	7.30 (1.56)			
	Affected	Control	5.32 (1.57)	−2.06	0.04*	−0.89 to −0.57
		Experimental	5.86 (1.25)			
EO (mm)	Healthy	Control	4.54 (1.35)	0.01	0.99	−0.66 to 0.65
		Experimental	4.54 (1.20)			
	Affected	Control	4.27 (1.30)	0.12	0.90	−0.73 to 0.64
		Experimental	4.23 (1.36)			
MF‐CSA (cm^2^)	Healthy	Control	5.47 (1.00)	1.68	0.09	−1.02 to 0.08
		Experimental	5.93 (1.14)			
	Affected	Control	4.46 (0.98)	−2.69	0.01*	−0.77 to −0.29
		Experimental	5.50 (1.10)			
MF thickness (mm)	Healthy	Control	26.32 (3.16)	1.14	0.25	−0.77 to 2.85
		Experimental	25.28 (3.83)			
	Affected	Control	21.95 (3.14)	−2.18	0.03*	−2.53 to −1.14
		Experimental	22.25 (3.93)			

Abbreviations: 95% CI, 95% confidence interval; EO, external oblique; IO, internal oblique; MF CSA, multifidus cross‐sectional area; SD, standard deviation; TrA, transversus abdominis.

* Significant at *p* < 0.05.

**Table 7:** Independent *t*‐test results for mean differences in pain intensity, disability, and range of motion after treatment between experimental and control groups.

Variable	Group	Mean (SD)	*t*‐statistic	*p*‐value	95% CI (lower–upper)
Pain intensity	Control	4.33 (1.53)	3.54	< 0.001*	0.59 to 2.13
	Experimental	2.96 (1.44)			
Functional disability	Control	47.33 (9.98)	4.97	< 0.001*	7.83 to 18.36
	Experimental	34.23 (10.39)			
Flexion ROM (°)	Control	35.86 (10.24)	−6.15	< 0.001*	−11.02 to −6.05
	Experimental	37.41 (9.73)			
Extension ROM (°)	Control	14.11 (4.36)	−3.07	0.01*	−3.85 to −0.81
	Experimental	15.84 (5.12)			

Abbreviations: 95% CI, 95% confidence interval; ROM, range of motion; SD, standard deviation.

* Significant at *p* < 0.05.

## 4 Discussion

This study evaluated the impact of stabilization exercises on trunk muscle function and clinical symptoms in patients with unilateral lumbar disc protrusion.

### 4.1 Pain Intensity, Functional Disability, and Lumbar ROM

These results are consistent with Ahmed et al., who found that although both physiotherapy and stabilization exercises reduced pain and improved function, stabilization training had a stronger effect. Similarly, other studies have reported greater improvements with stabilization exercises compared to routine therapy in managing pain and disability in Low back pain (LBP) patients [10, 28, 29].

As pain often limits joint mobility, both treatment programs were accompanied by decreases in pain and subsequent improvements in ROM. However, the experimental group demonstrated lower posttreatment pain intensity (2.96 ± 1.44 vs. 4.33 ± 1.53; *p* < 0.001) and descriptively greater ROM values, potentially pointing toward an empirical advantage of incorporating stabilization exercises, though formal interaction testing is required to confirm this added value (Table 7).

### 4.2 Stabilizer Muscle Thickness

In the control group, no significant changes were observed in trunk muscle dimensions at rest (*p* = 0.07–0.75). However, significant changes were observed during contraction on the affected side, particularly in the transversus abdominis, internal oblique, and multifidus muscles. This finding supports earlier studies by Critchley et al. and Sweeney et al., which also reported limited changes at rest but improved muscle performance during contraction in LBP patients compared to healthy individuals [30, 31].

Thus, conventional physiotherapy alone had no significant effect on resting muscle dimensions but was associated with significant alterations in muscle thickness during contraction in TrA, IO, MF‐AP, and multifidus cross‐sectional area (MF‐CSA) on the affected side (Table 3).

Inhibition of deep stabilizing muscles is common in LBP [32]. The observed changes in muscle dimensions during contraction suggest that pain reduction after 8 weeks of physiotherapy may have facilitated better muscle engagement during tasks such as abdominal hollowing and prone arm lifts [33].

Following the intervention, the experimental group demonstrated notable improvements in resting dimensions for the transversus abdominis, internal oblique, and lumbar multifidus cross‐sectional area bilaterally (Table 4). These findings align with reports by Hides et al. and Ashiyat et al., reinforcing the clinical value of targeted stabilization protocols in addressing deep stabilizer deficits [34, 35].

Furthermore, a significant increase in psoas major thickness was captured specifically on the symptomatic side (Table 5), a trend consistent with the observations of Kim et al. regarding core stabilization outcomes [36]. It is important to note that we cannot definitively claim that 8 weeks of regular physiotherapy combined with stability exercises has led to hypertrophy of the muscles in question. These findings should be interpreted with caution, as ultrasound‐based measurements cannot distinguish between true hypertrophy, changes in neuromuscular activation, or reductions in muscle spasm. Therefore, these changes are better considered indirect indicators of potential neuromuscular adaptations or adjustments in muscle activation patterns rather than definitive structural adaptations.

While both groups improved clinically, the descriptive increases in resting muscle dimensions within the experimental group visually parallel the intervention, though formal interaction testing is required to statistically confirm this added value.

Hides et al. emphasized that multifidus dysfunction may persist even after pain subsides, highlighting the importance of continued stabilization exercises to prevent recurrent LBP [37]. After intervention, the experimental group showed significant changes in muscle dimensions during contraction on both sides (except external oblique [EO]), whereas the control group showed changes only on the affected side. This supports the role of stabilization exercises in improving bilateral muscle performance during contraction. EO may have shown no change due to its limited stabilizing role [38].

Several studies have documented atrophy of the multifidus and psoas major muscles in patients with LBP [9, 39]. For instance, in 2007, a study by Kamaz et al. used CT scans to examine 36 patients with LBP and 34 healthy individuals, and reported that 80% of those with LBP exhibited atrophy of the multifidus and psoas muscles. The research revealed that the CSA of the psoas major, multifidus, paraspinal, and quadratus lumborum muscles was smaller in individuals with LBP compared to healthy controls [40].

Our findings indicate that the inclusion of stabilization exercises was accompanied by significant within‐group changes in trunk stabilizer dimensions (Tables 5 and 6), which occurred alongside parallel improvements in pain and functional disability (Table 7). However, since a formal time‐by‐group interaction matrix was not executed, these parallel outcomes must be interpreted strictly as co‐existing clinical trends rather than a confirmed mechanistic interplay directly driven by the intervention.

Changes in muscle thickness in the control group were limited to contraction on the affected side, whereas the experimental group demonstrated bilateral and resting changes. These findings may reflect potential functional or neural changes; however, they should be interpreted with caution, as reductions in muscle spasm may also contribute to the observed differences.

## 5 Conclusion

Both types of treatment (routine physiotherapy and routine physiotherapy combined with stabilization exercises) may help improve symptoms in patients with lumbar disc protrusion. However, the addition of stabilization exercises was associated with greater improvements.

While modifications in muscle dimensions during rest and contraction were observed alongside clinical improvements in the experimental group, the exact underlying mechanisms remain speculative. These changes may reflect a combination of altered neuromuscular activation, localized structural adaptations, or reductions in muscle spasm, rather than definitive evidence of muscular hypertrophy. Given the descriptive nature of our statistical approach, these parallel improvements cannot be interpreted as a direct causal relationship, and the findings should be viewed with appropriate clinical caution.

From a clinical standpoint, implementing stabilization exercises in daily physiotherapy practice may improve patient outcomes and reduce reliance on passive modalities in clinical practice for individuals with lumbar disc protrusion.

To gain more accurate insights and better differentiate between muscle spasm and contraction, further studies are needed to incorporate electromyography (EMG) to assess muscle activity more directly.

### 5.1 Limitations and Suggestions

A key limitation relates to the blinding procedure. Although outcome assessors were effectively blinded, the treating physiotherapist and participants could not be blinded due to the practical nature of supervised exercise interventions. This lack of blinding may have introduced performance and expectation biases, meaning that the observed improvements should be interpreted with caution, as they may partly reflect contextual or behavioral expectations rather than purely mechanical intervention effects.

Another limitation was the lack of psoas thickness measurement during contraction. Future research should include EMG for better assessment of muscle activation, use MRI to track fat infiltration, and incorporate follow‐up assessments to evaluate long‐term effects. More frequent assessments (e.g., at 4 and 8 weeks) are also recommended for a more detailed analysis.

In addition, the relatively small sample size may limit the generalizability of the findings.

Most importantly, a critical limitation of this study is the reliance on multiple paired and independent *t*‐tests instead of a multi‐factor mixed‐design analysis of variance (ANOVA). Consequently, the lack of formal group‐by‐time interaction testing remains an unresolved constraint, and the possibility of inflated type I error rates cannot be entirely ruled out. Therefore, the statistical significance of the between‐group differences should be viewed as preliminary, and future fully‐powered trials utilizing mixed ANOVA frameworks are strictly required to confirm these potential interaction effects.

## Clinical Relevance

- Adding stabilization exercises to routine physiotherapy was associated with greater clinical improvements in patients with unilateral lumbar disc protrusion.
- Ultrasonographic findings indicate changes in trunk muscle thickness during rest and contraction.
- This combined intervention can be readily implemented in outpatient physiotherapy settings.

## Author Contributions

**Morteza Taghipour:** conceptualization, data curation, methodology, writing – original draft, resources. **Nahid Rahmani:** conceptualization, methodology, writing – review and editing, validation. **Mohammad Ali Mohseni Bandpei:** conceptualization, methodology, writing – review and editing, supervision, project administration. **Fatemeh Rajabzadeh:** data curation, writing – original draft. **Iraj Abdollahi:** formal analysis, software. **Azadehsadat Mirtaleb:** data curation, formal analysis.

## Funding

The authors have nothing to report.

## Ethics Statement

This study was approved by the Ethics Committee of the University of Social Welfare and Rehabilitation Sciences (Approval Code: IR. USWR. REC.1396.211).

## Conflicts of Interest

The authors declare no conflicts of interest.

## Declaration of Generative AI and AI‐Assisted Technologies in the Writing Process

During the preparation of this manuscript, the authors used ChatGPT to improve language clarity and readability. The authors reviewed and edited the content as needed and take full responsibility for the content of the publication.

## Transparency Statement

Mohammad Ali Mohseni Bandpei affirms that this manuscript is an honest, accurate, and transparent account of the study being reported; that no important aspects of the study have been omitted; and that any discrepancies from the study as planned have been explained.

## Data Availability

The data that support the findings of this study are available on request from the corresponding author. The data are not publicly available due to privacy or ethical restrictions.
